# Association of Titin-Truncating Genetic Variants With Life-threatening Cardiac Arrhythmias in Patients With Dilated Cardiomyopathy and Implanted Defibrillators

**DOI:** 10.1001/jamanetworkopen.2019.6520

**Published:** 2019-06-28

**Authors:** Ben Corden, Julian Jarman, Nicola Whiffin, Upasana Tayal, Rachel Buchan, Joban Sehmi, Andrew Harper, William Midwinter, Karen Lascelles, Vias Markides, Mark Mason, John Baksi, Antonis Pantazis, Dudley J. Pennell, Paul J. Barton, Sanjay K. Prasad, Tom Wong, Stuart A. Cook, James S. Ware

**Affiliations:** 1 National Heart and Lung Institute, Imperial College London, London, United Kingdom; 2 Cardiovascular Research Centre, Royal Brompton and Harefield NHS Foundation Trust, London, United Kingdom; 3 Medical Research Council, London Institute for Medical Sciences, Imperial College London, London, United Kingdom; 4 National Heart Centre Singapore, Singapore

## Abstract

**Importance:**

There is a need for better arrhythmic risk stratification in nonischemic dilated cardiomyopathy (DCM). Titin-truncating variants (TTNtvs) in the *TTN* gene are the most common genetic cause of DCM and may be associated with higher risk of arrhythmias in patients with DCM.

**Objective:**

To determine if TTNtv status is associated with the development of life-threatening ventricular arrhythmia and new persistent atrial fibrillation in patients with DCM and implanted cardioverter defibrillator (ICD) or cardiac resynchronization therapy defibrillator (CRT-D) devices.

**Design, Setting, and Participants:**

This retrospective, multicenter cohort study recruited 148 patients with or without TTNtvs who had nonischemic DCM and ICD or CRT-D devices from secondary and tertiary cardiology clinics in the United Kingdom from February 1, 2011, to June 30, 2016, with a median (interquartile range) follow-up of 4.2 (2.1-6.5) years. Exclusion criteria were ischemic cardiomyopathy, primary valve disease, congenital heart disease, or a known or likely pathogenic variant in the lamin A/C gene. Analyses were performed February 1, 2017, to May 31, 2017.

**Main Outcome and Measures:**

The primary outcome was time to first device-treated ventricular tachycardia of more than 200 beats/min or first device-treated ventricular fibrillation. Secondary outcome measures included time to first development of persistent atrial fibrillation.

**Results:**

Of 148 patients recruited, 117 adult patients with nonischemic DCM and an ICD or CRT-D device (mean [SD] age, 56.9 [12.5] years; 76 [65.0%] men; 106 patients [90.6%] with primary prevention indications) were included. Having a TTNtv was associated with a higher risk of receiving appropriate ICD therapy (shock or antitachycardia pacing) for ventricular tachycardia or fibrillation (hazard ratio [HR], 4.9; 95% CI, 2.2-10.7; *P* < .001). This association was independent of all covariates, including midwall fibrosis measured by late gadolinium enhancement on cardiac magnetic resonance images (adjusted HR, 8.3; 95% CI, 1.8-37.6; *P* = .006). Having a TTNtv was also associated with the risk of receiving a shock (HR, 3.6; 95% CI, 1.1-11.6; *P* = .03). Individuals with a TTNtv and fibrosis had a greater rate of receiving appropriate device therapy than those with neither (HR, 16.6; 95% CI, 3.5-79.3; *P* < .001). Having a TTNtv was also a risk factor for developing new persistent atrial fibrillation (HR, 3.9; 95% CI, 1.3-12.0; *P* = .01).

**Conclusions and Relevance:**

Having a TTNtv was an important risk factor for clinically significant arrhythmia in patients with DCM and ICD or CRT-D devices. Having a TTNtv, especially in combination with midwall fibrosis confirmed with cardiovascular magnetic resonance imaging, may provide a risk stratification approach for evaluating the need for ICD therapy in patients with DCM. This hypothesis should be tested in larger studies.

## Key Points

**Question:**

Are titin-truncating variants in the *TTN* gene associated with life-threatening ventricular arrhythmias in patients with nonischemic dilated cardiomyopathy and an implanted cardioverter defibrillator or cardiac resynchronization therapy defibrillator?

**Findings:**

In this multicenter cohort study of 117 adult patients with dilated cardiomyopathy, 13 of 28 patients with titin-truncating variants (46%) received 1 or more appropriate implanted cardioverter defibrillator therapies compared with 13 of 89 patients without titin-truncating variants (16%), a significant difference.

**Meaning:**

Having a titin-truncating variant may be an independent risk factor for arrhythmia in patients with dilated cardiomyopathy and an implanted cardioverter defibrillator or cardiac resynchronization therapy defibrillator.

## Teaser

This cohort study investigates the association of titin-truncating genetic variants with life-threatening ventricular arrhythmias in adult patients with dilated cardiomyopathy who have implanted cardiac defibrillators or cardiac resynchronization therapy defibrillators.

## Introduction

Nonischemic dilated cardiomyopathy (DCM) is associated with potentially life-threatening ventricular arrhythmia,^1^ for which an implanted cardioverter defibrillator (ICD) may be lifesaving.^2^ However, ICD insertion carries risks, and many who receive a device will not benefit.^3,4^ Therefore, there is a pressing need for better prediction of arrhythmic risk in this population.^4^

Accessible and affordable sequencing technologies and improved variant annotation make the genetic assessment of patients with inherited cardiac conditions, such as DCM, more common in clinical practice and provide a platform for genetic stratification of patient treatment.^5^ Titin-truncating variants (TTNtvs) in the *TTN* gene are the most common genetic cause of DCM, accounting for approximately 15% of cases.^6^ Titin is the largest human protein and a crucial component of all striated muscle, in which it has structural, sensory, and signaling functions.^7^ Titin can be considered to be a molecular bidirectional spring, contributing to the contraction and relaxation of striated muscle, with additional roles in sarcomere organization, force transmission and transduction, and signaling responses.^7^

Our group has previously shown that patients with TTNtv-associated DCM are more likely to have a clinical history of arrhythmia (composite of atrial and ventricular arrhythmia, including nonsustained ventricular tachycardia [NSVT]) at the time of diagnosis.^8^ However, whether this is associated with an increased risk of atrial arrhythmia, ventricular arrhythmia, or both is not clear to our knowledge. In a 2017 cohort study^9^ of ambulant patients with DCM, we did not demonstrate an increased risk of new ventricular arrhythmia in patients with TTNtvs, although the total number of arrhythmic events in this low-risk group was small, limiting power to detect differences between groups. A 2018 study^10^ reported an association of incident ventricular arrhythmia with TTNtvs in a cardiomyopathy registry, particularly in patients with an additional environmental trigger. However, the analyses did not discriminate effects of potential covariates, such as ejection fraction or midwall fibrosis as measured by late gadolinium enhancement (LGE) on cardiovascular magnetic resonance images (CMR) and did not report on the incidence of new atrial fibrillation (AF). Therefore, whether TTNtvs are associated with an increased risk of clinically important or potentially life-threatening arrhythmia remains an important question. To answer this, we studied a group of patients with DCM and implanted devices (ICDs or cardiac resynchronization therapy defibrillators [CRT-Ds]; 90.6% implanted for primary prevention indications), allowing us to capture complete, 24-hour arrhythmia data for all patients over a long follow-up. We hypothesized that TTNtvs are associated with the occurrence of potentially life-threatening ventricular tachycardia (VT), defined as a heart rate of more than 200 beats/min, per the Multicenter Automatic Defibrillator Implantation Trial–Reduce Inappropriate Therapy study high-rate group,^11^ or ventricular fibrillation (VF), resulting in appropriate ICD therapy. A second hypothesis was that TTNtvs are associated with the development of persistent AF.

Midwall fibrosis, as measured by LGE on CMR, is a powerful predictor of arrhythmic events in DCM.^12^ Therefore, in a preplanned subgroup of patients with CMR data, a further aim was to examine whether information on TTNtv status was independent from fibrosis in assessing arrhythmic risk.

## Methods

### Participant Recruitment and Baseline Assessment

Adult patients 18 years and older with TTNtvs who had a diagnosis of primary DCM and an ICD or CRT-D were selected from the National Institute for Health Research Royal Brompton Biobank cohort. These were compared with adult patients with primary DCM who did not have TTNtvs selected randomly from the biobank (eAppendix 1 in the Supplement). Exclusion criteria were ischemic cardiomyopathy, primary valve disease, congenital heart disease, or a known or likely pathogenic variant in the lamin A/C gene. The study cohort included patients from 14 centers. None of the patients were related. All patients provided written informed consent. The study was approved by the South Central–Hampshire B research ethics committee and complied with the Declaration of Helsinki.^13^ Recruitment was from February 1, 2011, to June 30, 2016. Analyses were performed February 1, 2017, to May 31, 2017. This study is reported following the Strengthening the Reporting of Observational Studies in Epidemiology (STROBE) reporting guideline.

Dilated cardiomyopathy phenotype was confirmed wherever possible with CMR; when this was not possible (eg, owing to an ICD or CRT-D being in situ at recruitment), echocardiography was used. For patients who underwent CMR, DCM criteria were raised left ventricle (LV) end-diastolic volume and reduced LV ejection fraction (LVEF), with reference to age-predicted and sex-adjusted nomograms.^14^ Echocardiography criteria were LV end-diastolic diameter more 117% of the age- and body surface area–predicted value^15^ and LVEF less than 45% of the predicted value. Absence of ischemic etiology, primary valve disease, and congenital or structural heart disease were confirmed by review of coronary angiograms, noninvasive imaging, and clinical history. Patients with DCM and a known or likely pathogenic variant in the lamin A/C gene were excluded from the cohort, as DCM associated with lamin A/C is already known to present with a severe arrhythmogenic phenotype, as reflected in international ICD guidelines.^16,17^

Interrogation data on ICD and CRT-D devices, including electrograms for all events, were sourced for each patient from every center where they had undergone device interrogation. Patients with missing data were excluded. Baseline characteristics were not significantly different between the final cohort compared with those excluded owing to missing arrhythmia data (eTable 1 in the Supplement), and tipping point analyses showed that the main findings were robust to extreme results among those with missing arrhythmia data (eFigure 1 in the Supplement).

### Genetic Analysis

All patients underwent targeted next-generation sequencing at our core facility to detect likely or known pathogenic TTNtvs, with variant interpretation according to international guidelines, as previously described.^6^ Full details of the variants tested and detected can be found in eAppendix 2, eTable 2, and eTable 3 in the Supplement.

### Follow-up and Adjudication of Arrhythmia Outcomes

Follow-up was truncated at 9 years, given the small number of patients who had follow-up data beyond this time. All ICD and CRT-D interrogation data were reviewed from device implantation until either the most recent follow-up or the follow-up immediately prior to 9 years after implantation, whichever came first. Three of us (B.C., J.J., and T.W.) reviewed the interrogation data and were blinded to genotype throughout. The primary end point was time to first appropriate ICD therapy (antitachycardia pacing or shock) for VT or VF. Secondary end points were time to first appropriate ICD shock and time to first episode of persistent AF (defined as AF for ≥7 days or requiring cardioversion). Appropriateness of therapy was verified independently by 3 of us (B.C., J.J., and T.W.), blinded to genotype, who manually inspected device electrograms for all events.

### Statistical Analysis

Unless otherwise stated, statistical analyses were performed using R statistical software version 3.3.2 (R Project for Statistical Computing). Categorical data were compared using Fisher exact tests. Differences between continuous variables were assessed with *t* tests or Mann-Whitney *U* tests, as appropriate.

Kaplan-Meier plots were constructed for the primary and secondary end points according to TTNtv status. If a patient did not experience an arrhythmic event, data were right censored on the day of their most recent device interrogation. Statistically significant differences were assessed by log-rank tests. *P* values were 2-tailed and statistical significance was set at less than .05. Hazard ratios (HRs) for log-rank tests are reported with 95% CIs constructed using the method by Lin et al,^18^ implemented in SAS Studio statistical software version 3.6 (SAS Institute). Cox proportional hazard models were constructed to adjust for potentially confounding variables. The proportional hazards assumption was verified using Schoenfield residuals.

## Results

There were 37 patients with DCM and TTNtvs in the biobank. They were compared with 111 (3 × 37) patients with DCM who did not have TTNtvs, selected randomly from the biobank (eAppendix 1 in the Supplement) in the initial cohort. Nine patients with TTNtvs and 22 patients who did not have TTNtvs were excluded because of missing or destroyed records, resulting in a final study population of 117 patients (28 patients with TTNtvs [23.9%]; 89 patients without TTNtvs [76.1%]). Mean (SD) age at implant was 56.9 (12.5) years, 76 patients (65.0%) were men, and 106 patients (90.6%) had primary prevention indications. Table 1 gives baseline characteristics for the patient cohorts; race/ethnicity were self-reported. Median (interquartile range [IQR]) follow-up was 4.2 (2.1-6.5) years.

**Table 1.** Characteristics of Cohort

Characteristic	Group, Mean (SD)		*P* Value^a^
	With TTNtvs (n = 28)	Without TTNtvs (n = 89)	
DCM diagnosed via CMR, No.^b^	21	69	NA
DCM diagnosed via echocardiography, No.^c^	7	20	NA
Age at implant, y	51.1 (10.8)	58.7 (12.5)	.005
Men, No. (%)	24 (86)	52 (58)	.01
Primary prevention indication, No. (%)	25 (89)	81 (91)	.72
CMR measurements^b^			
LVEF, %	31.2 (10.7)	30.5 (10.2)	.79
RVEF, %	45.4 (13.6)	48 (16.2)	.51
LV EDV/BSA, mL/m^2^	140 (31.1)	144 (36.6)	.68
RV EDV/BSA, mL/m^2^	87.4 (28.6)	85.3 (28.6)	.78
Midwall fibrosis, No. (%)	13 (62)	28 (41)	.13
Echocardiographic measurements^c^			
LVEF, %	27 (10.6)	26.1 (9.2)	.79
LV EDD/BSA, mm/m^2^	31.6 (5.1)	34.1 (4.7)	.17
Follow-up, median (IQR), y	3.8 (2.4-5.4)	4.4 (2.1-7.0)	.65
Time from diagnosis to device implant, median (IQR), y	1.2 (0.3-4.8)	1.0 (0.2-4.4)	.88
LBBB at implant, No. (%)	7 (25)	57 (64)	<.001
CRT, No. (%)	11 (39)	65 (73)	.003
BMI	28.4 (6.3)	27.9 (5.5)	.66
Race/ethnicity, No. (%)			
White	25 (89.2)	77 (86.5)	>.99
Black	2 (7.1)	4 (4.5)	.63
Asian	1 (3.6)	5 (5.6)	>.99
Other or mixed	0	3 (3.4)	>.99
Medications, No. (%)			
ACE inhibitor/ARB	28 (100)	88 (98.9)	>.99
β-Blocker	25 (89.2)	72 (80.9)	.40
Mineralocorticoid-receptor antagonist	20 (71.4)	61 (68.5)	.82
Amiodarone	6 (21.4)	19 (21.4)	>.99

Abbreviations: ACE, angiotensin-converting enzyme; ARB, angiotensin II receptor blocker; BMI, body mass index (calculated as weight in kilograms divided by height in meters squared); BSA, body surface area; CMR, cardiovascular magnetic resonance; CRT, cardiac resynchronization therapy; DCM, dilated cardiomyopathy; EDD, end-diastolic diameter; EDV, end-diastolic volume; EF, ejection fraction; IQR, interquartile range; LBBB, left bundle branch block; LV, left ventricle; NA, not applicable; RV, right ventricle; TTNtv, titin-truncating variant.

^a^ Categorical variables compared with Fisher exact tests and continuous variables with *t* tests or Mann-Whitney *U* tests.

^b^ In patients for whom DCM diagnosis was confirmed with CMR, CMR data are included.

^c^ In patients for whom DCM diagnosis was confirmed with echocardiography, echocardiographic data are included.

### Association of TTNtvs With Appropriate Defibrillator Therapy

**Figure 1.**
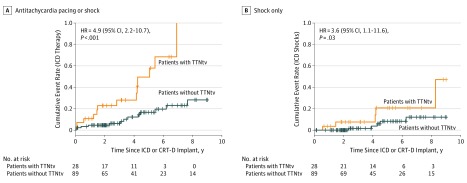
Associations of Titin-Truncating Variants (TTNtvs) With Implanted Cardioverter Defibrillator (ICD) Therapy for Ventricular Tachycardia or Ventricular Fibrillation Kaplan-Meier plots for appropriate ICD therapy (antitachycardia pacing or shock) (A) or appropriate shock for ventricular tachycardia more than 200 beats/min or ventricular fibrillation (B). Crosses indicate censoring; CRT-D, cardiac resynchronization therapy defibrillator; and HR, hazard ratio.

Patients with TTNtvs had a significantly greater risk of receiving an appropriate ICD therapy compared with those without TTNtvs (HR, 4.9; 95% CI, 2.2-10.7; *P* < .001) (Figure 1). Overall, 13 of 28 patients with TTNtvs (46%) received 1 or more appropriate ICD therapies over a median (IQR) follow-up of 3.8 (2.4-5.4) years compared with 13 of 89 patients without TTNtvs (15%) over a median (IQR) follow-up of 4.4 (2.1-7.0) years. Restricting the analysis to ICD shocks (ie, discounting antitachycardia pacing), 5 patients with TTNtvs (18%) received 1 or more appropriate shocks compared with 5 patients without TTNtvs (6%) (HR, 3.6; 95% CI, 1.1-11.6; *P* = .03) (Figure 1).

### Association of TTNtvs With Appropriate ICD Therapy in Patients With a Primary Prevention Indication

A small number of the cohort had a secondary prevention indication for an ICD or a CRT-D (3 patients with TTNtvs [11%]; 8 patients without TTNtvs [9%]; *P* = .72), which may be a group with a different baseline risk of arrhythmia. Therefore, we repeated the analyses separately for those with a primary vs a secondary prevention indication. The pattern of results was unchanged. For patients with a primary prevention indication, the HR for any ICD therapy was 4.9 (95% CI, 2.1-11.5; *P* < .001). For appropriate shock alone, the HR was 5.1 (95% CI, 1.5-18.0; *P* = .008) (eFigure 2 in the Supplement). For the 11 patients with secondary prevention indications, there was no statistically significant association of TTNtv with ICD therapy (HR, 2.8; 95% CI, 0.4-20.1; *P* = .29).

### Association of TTNtvs With Appropriate Device Therapy Independent of Covariates

As shown in Table 1, the groups differed in terms of age, sex, the proportion with left bundle branch block (LBBB), and the proportion receiving resynchronization therapy in addition to a defibrillator. Therefore, multivariable Cox proportional hazard models were constructed containing these variables in addition to TTNtv status. Titin-truncating variants remained the only variable significantly associated with ICD therapy in both the whole cohort (adjusted HR, 3.5; 95% CI, 1.3-9.5; *P* = .01) and the primary prevention cohort (adjusted HR, 3.6; 95% CI, 1.1-11.7; *P* = .03). Additional adjustment for LVEF and body mass index did not alter this result (eTables 4-7 in the Supplement).

Nonsustained VT is known to predict sustained ventricular arrhythmias,^1^ and we have previously shown that patients with TTNtvs are more likely to have a history of NSVT at the time of DCM diagnosis.^6^ In this study, we found that a history of NSVT was associated with ICD therapy (HR, 4.7; 95% CI, 1.6-13.8; *P* = .001). Therefore, a further model was constructed with history of NSVT prior to device implantation as an additional covariate. Titin-truncating variants remained significantly associated with ICD therapy, independently of a history of NSVT and other covariates (adjusted HR, 3.1; 95% CI, 1.1-9.2; *P* = .04) (eTable 8 in the Supplement). In equivalent models for appropriate ICD shock, no variables were significantly and independently associated with ICD therapy (eTables 9-11 in the Supplement).

### Association of TTNtvs With Appropriate ICD Therapy Independent of Midwall Fibrosis

Data on LGE from CMR were available for 90 patients (21 patients with TTNtvs [75%]; 69 patients without TTNtvs [78%]) (eTable 12 in the Supplement). In this subgroup, 13 of 21 patients with TTNtvs (61.9%) had evidence of midwall fibrosis as assessed by LGE on CMR vs 28 of 69 patients without TTNtvs (40.6%) (odds ratio, 2.4; 95% CI, 0.78-7.5; *P* = .13). Midwall fibrosis was significantly associated with appropriate ICD therapy (HR, 3.6; 95% CI, 1.2-11.2; *P* = .02). Having a TTNtv remained the only variable independently associated with appropriate ICD therapy after adjusting for midwall fibrosis alone (adjusted HR, 7.9; 95% CI, 2.6-24; *P* < .001) and after adjusting for midwall fibrosis plus age, sex, LBBB, device type, body mass index, and LVEF (adjusted HR, 8.3; 95% CI, 1.8-37.6; *P* = .006) (Table 2). Restricting the analysis to the primary prevention cohort did not alter the results (eTable 13 in the Supplement).

**Table 2.** Association of TTNtv Status With Appropriate ICD Therapy After Adjustment for Multiple Covariates^a^

Variable	Adjusted HR (95% CI)	*P* Value
Age (per 5 y)	1.06 (0.80-1.36)	.74
Sex (male vs female)	0.94 (0.21-4.33)	.94
Device type (ICD vs CRT-D)	1.35 (0.37-4.97)	.65
LBBB	1.21 (0.30-4.82)	.79
LVEF (per 5%)	0.90 (0.72-1.13)	.39
BMI	0.98 (0.89-1.08)	.69
Midwall fibrosis (present vs absent)	2.14 (0.57-8.06)	.26
TTNtv (with vs without)	8.33 (1.85-37.61)	.006

Abbreviations: BMI, body mass index; CRT-D, cardiac resynchronization therapy defibrillator; HR, hazard ratio; ICD, implanted cardioverter defibrillator; LBBB, left bundle branch block; LVEF, left ventricle ejection fraction; TTNtv, titin-truncating variant.

^a^ Cox proportional hazards model for time to first appropriate ICD therapy (antitachycardia pacing or shocks). The association of TTNtv with ICD therapy remained after adjustment for age, sex, device type, presence of LBBB, LVEF, BMI, and the presence of midwall fibrosis as measured by late gadolinium enhancement on cardiovascular magnetic resonance images.

**Figure 2.**
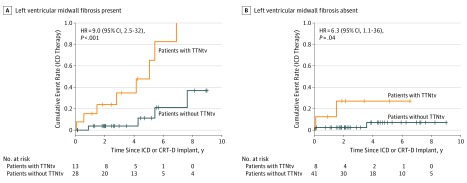
Associations of Titin-Truncating Variants (TTNtvs) and Midwall Fibrosis With Implanted Cardioverter Defibrillator (ICD) Therapy Kaplan-Meier plots for the time to first appropriate ICD therapy by TTNtv status and presence (A) or absence (B) of midwall fibrosis. Midwall fibrosis and TTNtvs are independently associated with appropriate ICD therapy and are additive. Of the 41 patients who did not have TTNtvs or midwall fibrosis, there were only 2 episodes of ICD therapy (5%) and no ICD shocks. In contrast, of the 13 patients who had TTNtvs and midwall fibrosis, there were 8 appropriate ICD therapies (62%) and 5 shocks (38%). Patients with both risk factors had a 16-fold increased hazard ratio (HR) of ICD therapy compared with patients with neither. Crosses indicate censoring; CRT-D, cardiac resynchronization therapy defibrillator.

Figure 2 shows Kaplan-Meier plots split by TTNtv and midwall fibrosis status. Of note, the risk of ventricular arrhythmia was very low in patients with neither a TTNtv nor evidence of midwall fibrosis on their CMR results compared with a high risk in those with both. Of the 41 patients without a TTNtv or midwall fibrosis, only 2 (5%) had episodes of ICD therapy and none had ICD shocks. In contrast, of the 13 patients with TTNtvs and midwall fibrosis, 8 (62%) received appropriate ICD therapies and 5 (38%) received shocks. The HR of receiving an appropriate ICD therapy among patients with TTNtvs and midwall fibrosis vs patients without TTNtvs or midwall fibrosis was 16.6 (95% CI, 3.5-79.3; *P* < .001).

### Association of TTNtvs With Appropriate ICD Therapy in Patients With an ICD and Patients With a CRT-D

There were fewer patients receiving cardiac resyncronization therapy in the TTNtv group (Table 1). As cardiac resynchronization therapy is associated with reduced risk of arrhythmia, this is a potential source of bias. Therefore, we repeated the primary analysis separately for patients with ICDs and CRT-Ds. The association of TTNtvs with appropriate defibrillator therapy was significant for patients with an ICD (HR, 4.0; 95% CI, 1.2-13.3; *P* = .01) and patients with a CRT-D (HR, 3.6; 95% CI, 1.2-11.4; *P* = .03), with a similar size of effect. Consistent with this, there was no statistical interaction between device type and TTNtvs in a Cox model (HR, 0.83; 95% CI, 0.2-4.8; *P* =. 96).

### Association of TTNtvs With the Development of Persistent AF

At device implant, 6 patients with TTNtvs (21%) were in permanent AF compared with 10 patients without TTNtvs (11%) (odds ratio, 2.1; 95% CI, 0.7-6.6; *P* = .21). Of patients in sinus rhythm at implant, 6 with TTNtvs (27%) developed persistent AF over a median (IQR) follow-up of 3.7 (2.2-6.2) years compared with 7 without TTNtvs (9%) over a median (IQR) follow-up of 4.5 (2.1-6.8) years (HR, 3.9; 95% CI, 1.3-12.0; *P* = .01). In a Cox model, TTNtv status remained associated with new-onset persistent AF after adjusting for age, sex, LBBB, device type, body mass index, and LVEF (HR, 9.2; 95% CI, 2.0-43.0; *P* = .005).

### Association of TTNtvs With Inappropriate ICD Therapy

There was no significant association of TTNtv status with time to first inappropriate ICD therapy (HR, 2.3; 95% CI, 0.9-6.1; *P* = .10) or shock (HR, 1.9; 95% CI, 0.7-5.2; *P* = .25) (eFigure 3 in the Supplement). The most common cause of inappropriate therapy was AF (9 incidents of any ICD therapy; 8 incidents of shock), followed by other atrial arrhythmia (7 incidents of any therapy; 5 incidents of shock) and lead failure (2 incidents of any therapy; 3 incidents of shock).

## Discussion

We found that TTNtvs, the most prevalent genetic cause of DCM, were associated with a significantly higher risk of appropriate ICD therapy for potentially life-threatening ventricular arrhythmias. This association was independent of other known predictors of ventricular arrhythmia, including age, sex, LVEF, history of NSVT, and the presence of LV fibrosis.

Our previous study^8^ showed an increased prevalence of a history of arrhythmia (composite of atrial and ventricular arrhythmia) in TTNtv-associated DCM at the time of recruitment. Our data show that, for those meeting criteria for ICD implantation, this translates into a higher risk of incident life-threatening arrhythmia requiring ICD therapy, including shocks.

### Mechanisms

Myocardial fibrosis is a substrate for ventricular arrhythmia in DCM, and midwall fibrosis is associated with sudden death and appropriate ICD therapy.^12,19^ We confirmed the association of midwall fibrosis with device therapy but found no significant difference in the prevalence of fibrosis among patients with TTNtvs vs those without, consistent with previous work.^6^ Furthermore, we found that the association of TTNtvs with ventricular arrhythmias was independent of the presence of midwall fibrosis, suggesting that the association of TTNtvs with ventricular arrhythmia susceptibility is not mediated via midwall fibrosis. However, we note that LGE on CMR is not a sensitive marker for diffuse interstitial fibrosis, which may have differed between the groups. Interestingly, a 2018 study^10^ found that patients with DCM and TTNtvs had both an increased risk of ventricular arrhythmia and increased interstitial fibrosis on ventricular biopsy compared with patients with DCM who did not have TTNtvs. In future studies, this association could be explored noninvasively via CMR T1 mapping.

We found that the combination of TTNtvs with midwall fibrosis was associated with particularly high risk of ventricular arrhythmia, whereas the absence of both was associated with a very low risk. Therefore, combining knowledge of TTNtv with fibrosis status has potential to aid risk stratification when deciding on the need for an ICD in patients with DCM. If replicated in larger observational studies, this hypothesis could be tested in prospective studies.

In our study, arrhythmic risk did not result from more severe ventricular impairment or dilatation in patients with TTNtvs compared with patients without TTNtvs, consistent with prior work.^6,16,20^ However, our group has previously shown that compared with hearts of patients who do not have TTNtvs, the hearts of patients with TTNtvs and DCM have thinner LV walls and lower indexed LV mass for the same degree of dilatation, a combination resulting in higher LV wall stress.^6^ Increased wall stress is associated with greater risk of ventricular arrhythmia both through promotion of triggered activity (via early and late afterdepolarizations) and through facilitation of reentry circuits (shortening the effective refractory period with increased dispersion),^21,22^ providing potential mechanistic explanations for our observations.

Consistent with 2 reports from 2017,^16,20^ we observed that patients with TTNtvs were less likely to have LBBB and were therefore less likely to receive CRT along with their ICD. However, the association of TTNtv status with ventricular arrhythmias was unaffected by adjustment for LBBB and CRT prevalence, there was no statistical interaction between device type with TTNtv status, and there were significant and similarly sized associations of TTNtvs with ICD therapy when patients with ICD or CRT-D devices were analyzed separately. Therefore, the association of TTNtvs with device therapy was not changed by the addition of CRT.

### Atrial Fibrillation

Having a TTNtv was also associated with the development of persistent AF. To our knowledge, this is the first time an association with incident AF, and particularly with prolonged AF, has been demonstrated in patients with DCM.

Analogous to the response observed in ventricles of patients with TTNtvs,^6^ it may be that TTNtvs are associated with thinner atrial walls for a given degree of dilatation, resulting in higher atrial wall stress. This would cause greater atrial myocyte stretch, known to promote AF both through electrophysiological remodeling and the stimulation of atrial fibrosis.^23,24^

### Limitations

There are potential limitations to this work. Our study was carried out in patients with an ICD or a CRT-D, by definition a group at higher risk of arrhythmia; therefore, findings cannot be easily extrapolated to all patients with DCM. However, our results are relevant for patients meeting conventional indications for ICD or CRT-D therapy, especially for primary prevention, who composed 90.6% of our cohort. In addition, the small numbers of patients with secondary prevention indications and of female patients indicate that the results are difficult to extrapolate to these subpopulations.

Second, pacing records were accessed retrospectively, and while care was taken to collect all possible records, some were unobtainable. Although the baseline characteristics were similar across the groups, we cannot exclude a degree of bias.

Appropriate ICD therapies are not a perfect surrogate for sudden cardiac death, as some ventricular arrhythmias will self-resolve.^25^ This may be particularly true of antitachycardia pacing–terminated arrhythmia. However, it remains the case that ICDs reduce the risk of sudden cardiac death in DCM, which can only be due to their effect in terminating malignant arrhythmias, and we restricted our analysis to faster VT or VF that are most likely to have a significant hemodynamic impact.^3,11^ Nonetheless, although we hypothesize that TTNtvs are associated with risk of sudden cardiac death, at least in patients meeting the indication for a primary prevention defibrillator (eg, LVEF <35%), this remains to be formally assessed in large prospective studies.

A limitation of any retrospective study using ICD or CRT-D data is that it is not possible to standardize the approach to therapy for tachyarrhythmia to the same degree as would be possible in a prospective study, which may lead to bias. To mitigate this, we used a strict definition of arrhythmia using Multicenter Automatic Defibrillator Implantation Trial–Reduce Inappropriate Therapy study^11^ high-rate group criteria (sustained VT >200 beats/min or VF). Also, clinicians were blinded to genotype throughout the study, so knowledge of TTNtv status could not have systemically influenced device programming.

History of NSVT or AF prior to entering the study was ascertained from clinical notes. Therefore, the method of detecting these arrhythmias (eg, Holter monitoring or telemetry) as well as the frequency of monitoring varied among patients. Our study was powered to test our primary outcome measure, time to first appropriate ICD therapy for VT more than 200 beats/min or VF. It was not powered for other measures, such as time to shock or mortality, for which there were few events and results showed wide confidence intervals; therefore, these results should be interpreted with a degree of caution. In addition, the number of patients with TTNtvs (and the number of events) was relatively small, leading to the possibility of model overfitting. However, our central finding of increased risk of ventricular arrhythmia was statistically significant and robust across several subgroup analyses (primary vs secondary prevention indications, ICD vs CRT-D recipients) and after adjustment for multiple covariates.

Additionally, this is an observational study with relatively small numbers of participants. Although we have taken care to adjust for differences between the groups, some bias may remain. These results should be interpreted as hypothesis generating and should be replicated in larger observational and prospective studies.

## Conclusions

This study found that TTNtvs in the *TTN* gene were a risk factor for clinically relevant arrhythmia in patients with DCM and ICD or CRT-D devices. Knowledge of a patient’s TTNtv status may be complementary to midwall fibrosis imaging by CMR in predicting arrhythmic risk in patients with DCM.

## Notes

Supplement. **eAppendix 1.** Additional Methods **eAppendix 2.** Additional Results **eFigure 1.** Tipping-Point Analysis for the Association of Titin-Truncating Variants With Appropriate Implanted Cardioverter Defibrillator Therapy Is Robust to Extreme Results Amongst Patients With Missing Implanted Cardioverter Defibrillator Data **eFigure 2.** Association of Titin-Truncating Variants With an Increased Risk of Implanted Cardioverter Defibrillator Therapy in Analysis Restricted to Patients in the Primary Prevention Cohort **eFigure 3.** No Significant Difference in Inappropriate Implanted Cardioverter Defibrillator Therapy Among Patients With Titin-Truncating Variants vs Those Without **eTable 1.** Baseline Characteristics of Patients in the Study Cohort vs Those Excluded Owing to Incomplete or Missing Arrhythmia Data **eTable 2.** Details of Likely Pathogenic and Pathogenic Titin-Truncating Variants in the Cohort **eTable 3.** Details of Likely Pathogenic and Pathogenic Variants Found in the Cohort Other Than Titin-Truncating Variants in the *TTN* Gene **eTable 4.** Cox Proportional Hazards Model for Time to First Appropriate Implanted Cardioverter Defibrillator Therapy Among the Whole Cohort **eTable 5.** Cox Proportional Hazards Model for Time to First Appropriate Implanted Cardioverter Defibrillator Therapy Among the Primary Prevention Cohort **eTable 6.** Cox Proportional Hazards Model for Time to First Appropriate Implanted Cardioverter Defibrillator Therapy Among the Whole Cohort With Adjustment for Body Mass Index and Left Ventricle Ejection Fraction **eTable 7.** Cox Proportional Hazards Model for Time to First Appropriate Implanted Cardioverter Defibrillator Therapy Among the Primary Prevention Cohort With Adjustment for Body Mass Index and Left Ventricle Ejection Fraction **eTable 8.** Cox Proportional Hazards Model for Time to First Appropriate Implanted Cardioverter Defibrillator Therapy Among the Whole Cohort With Adjustment for History of Nonsustained Ventricular Tachycardia **eTable 9.** Cox Proportional Hazards Model for Time to First Appropriate Shock Among the Whole Cohort **eTable 10.** Cox Proportional Hazards Model for Time to First Appropriate Shock Among the Primary Prevention Cohort **eTable 11.** Cox Proportional Hazards Model for Time to First Appropriate Shock With Adjustment for History of Nonsustained Ventricular Tachycardia **eTable 12.** Baseline Characteristics of Patients With Cardiovascular Magnetic Resonance Imaging Data vs Those Without **eTable 13.** Cox Proportional Hazards Model for Time to First Appropriate Implanted Cardioverter Defibrillator Therapy Among the Primary Prevention Patient Cohort With Adjustment for Midwall Fibrosis as Determined by Late Gadolinium Enhancement on Cardiovascular Magnetic Resonance Images **eReferences**
