# Supplementary material for: Association of Titin-Truncating Genetic Variants With Life-threatening Cardiac Arrhythmias in Patients With Dilated Cardiomyopathy and Implanted Defibrillators
Source: JAMA Netw Open. 2019 Jun 28;2(6):e196520. doi: 10.1001/jamanetworkopen.2019.6520 (PMC6604081; doi:10.1001/jamanetworkopen.2019.6520)
Supplement: Supplement. — eAppendix 1. Additional Methods eAppendix 2. Additional Results eFigure 1. Tipping-Point Analysis for the Association of Titin-Truncating Variants With Appropriate Implanted Cardioverter Defibrillator Therapy Is Robust to Extreme Results Amongst Patients With Missing Implanted Cardioverter Defibrillator Data eFigure 2. Association of Titin-Truncating Variants With an Increased Risk of Implanted Cardioverter Defibrillator Therapy in Analysis Restricted to Patients in the Primary Prevention Cohort eFigure 3. No Significant Difference in Inappropriate Implanted Cardioverter Defibrillator Therapy Among Patients With Titin-Truncating Variants vs Those Without eTable 1. Baseline Characteristics of Patients in the Study Cohort vs Those Excluded Owing to Incomplete or Missing Arrhythmia Data eTable 2. Details of Likely Pathogenic and Pathogenic Titin-Truncating Variants in the Cohort eTable 3. Details of Likely Pathogenic and Pathogenic Variants Found in the Cohort Other Than Titin-Truncating Variants in the TTN Gene eTable 4. Cox Proportional Hazards Model for Time to First Appropriate Implanted Cardioverter Defibrillator Therapy Among the Whole Cohort eTable 5. Cox Proportional Hazards Model for Time to First Appropriate Implanted Cardioverter Defibrillator Therapy Among the Primary Prevention Cohort eTable 6. Cox Proportional Hazards Model for Time to First Appropriate Implanted Cardioverter Defibrillator Therapy Among the Whole Cohort With Adjustment for Body Mass Index and Left Ventricle Ejection Fraction eTable 7. Cox Proportional Hazards Model for Time to First Appropriate Implanted Cardioverter Defibrillator Therapy Among the Primary Prevention Cohort With Adjustment for Body Mass Index and Left Ventricle Ejection Fraction eTable 8. Cox Proportional Hazards Model for Time to First Appropriate Implanted Cardioverter Defibrillator Therapy Among the Whole Cohort With Adjustment for History of Nonsustained Ventricular Tachycardia eTable 9. Cox Proportional Hazards Model [file jamanetwopen-2-e196520-s001.pdf]

Supplementary Online Content

Corden B, Jarman J, Whiffin N, et al. Association of titin-truncating genetic variants with life-threatening cardiac arrhythmias in patients with dilated cardiomyopathy and implanted defibrillators. *JAMA Netw Open*. 2019;2(6):e196520.
doi:10.1001/jamanetworkopen.2019.6520

eAppendix 1. Additional Methods

eAppendix 2. Additional Results

eFigure 1. Tipping-Point Analysis for the Association of Titin-Truncating Variants With Appropriate Implanted Cardioverter Defibrillator Therapy Is Robust to Extreme Results Amongst Patients With Missing Implanted Cardioverter Defibrillator Data

eFigure 2. Association of Titin-Truncating Variants With an Increased Risk of Implanted Cardioverter Defibrillator Therapy in Analysis Restricted to Patients in the Primary Prevention Cohort

eFigure 3. No Significant Difference in Inappropriate Implanted Cardioverter Defibrillator Therapy Among Patients With Titin-Truncating Variants vs Those Without

eTable 1. Baseline Characteristics of Patients in the Study Cohort vs Those Excluded Owing to Incomplete or Missing Arrhythmia Data

eTable 2. Details of Likely Pathogenic and Pathogenic Titin-Truncating Variants in the Cohort

eTable 3. Details of Likely Pathogenic and Pathogenic Variants Found in the Cohort Other Than Titin-Truncating Variants in the *TTN* Gene

eTable 4. Cox Proportional Hazards Model for Time to First Appropriate Implanted Cardioverter Defibrillator Therapy Among the Whole Cohort

eTable 5. Cox Proportional Hazards Model for Time to First Appropriate Implanted Cardioverter Defibrillator Therapy Among the Primary Prevention Cohort

eTable 6. Cox Proportional Hazards Model for Time to First Appropriate Implanted Cardioverter Defibrillator Therapy Among the Whole Cohort With Adjustment for Body Mass Index and Left Ventricle Ejection Fraction

eTable 7. Cox Proportional Hazards Model for Time to First Appropriate Implanted Cardioverter Defibrillator Therapy Among the Primary Prevention Cohort With Adjustment for Body Mass Index and Left Ventricle Ejection Fraction

eTable 8. Cox Proportional Hazards Model for Time to First Appropriate Implanted Cardioverter Defibrillator Therapy Among the Whole Cohort With Adjustment for History of Nonsustained Ventricular Tachycardia

© 2019 Corden B et al. *JAMA Network Open.*

eTable 9. Cox Proportional Hazards Model for Time to First Appropriate Shock Among the Whole Cohort

eTable 10. Cox Proportional Hazards Model for Time to First Appropriate Shock Among the Primary Prevention Cohort

eTable 11. Cox Proportional Hazards Model for Time to First Appropriate Shock With Adjustment for History of Nonsustained Ventricular Tachycardia

eTable 12. Baseline Characteristics of Patients With Cardiovascular Magnetic Resonance Data vs Those Without

eTable 13. Cox Proportional Hazards Model for Time to First Appropriate Implanted Cardioverter Defibrillator Therapy Among the Primary Prevention Patient Cohort With Adjustment for Midwall Fibrosis as Determined by Late Gadolinium Enhancement on Cardiovascular Magnetic Resonance Images

eReferences

This supplementary material has been provided by the authors to give readers additional information about their work.

© 2019 Corden B et al. *JAMA Network Open.*

eAppendix 1. Additional Methods

Biobank recruitment

There were three routes to recruitment into the NIHR Royal Brompton Cardiovascular Biobank - patients were referred to the Royal Brompton Hospital or Harefield Hospital either for device (ICD/CRT-D) implantation, for cardiovascular magnetic resonance (CMR) imaging, or for review at the dedicated cardiomyopathy or heart failure clinics. The Biobank database includes demographic and clinical information collected via patient interview and clinical records, including the results of diagnostic tests. For the current study, subsequent clinical and device follow-up was then at Royal Brompton Hospital, Harefield Hospital or one of the following 12 centres: Basildon University Hospital, Bristol Heart Institute, Ealing Hospital, Kings College Hospital, Maidstone Hospital, Northwick Park Hospital, Royal Berkshire Hospital, Royal United Hospital Bath, St Helier University Hospital, Tunbridge Wells Hospital, Watford General Hospital and Wexham Park Hospital.

Control group selection

Before commencing data collection, we calculated that a population of 30 TTNtv +ve patients and 90 TTNtv-ve patients would give 80% power to detect a hazard ratio of ≥ 3.7 at the 5% level, assuming a probability of ICD therapy of 20% over the study period[1]. Power calculations showed that increasing the number of control subjects beyond ~3x the number of TTNtv+ve subjects led to only very minimal increases in power. For example, increasing the number of controls to 210 (7 x the number of TTNtv +ve patients) would increase our power to detect a hazard ratio ≥ 3.7 by only 5% (to 85%)[1], or reduce the hazard ratio detectable with 80% power from 3.7 to 3.4. Therefore, to balance statistical power with cost and time of collection and interpretation of electrogram data, we selected 111 control subjects (3 x the number of TTNtv +ve patients) from the Biobank, selected at random using a random number generator applied to a list of anonymised patient identifiers.

Formula for power calculations[1]

Power (1- Type II error) was determined using:

$$Power = \Phi\left(z - z_{1-\frac{\alpha}{2}}\right) + \Phi\left(-z - z_{1-\frac{\alpha}{2}}\right)$$

where

$$z = (\ln(\theta) - \ln(\theta_0))\sqrt{n\, pA\, pB\, pE}$$

and

θ is the hazard ratio

pE is the overall probability of the event occurring within the study period (taken as 20% for our calculations, based on previous studies[2-4])

pA and *pB* are the proportions of the sample size allocated to each group (e.g. in our example with 30 TTNtv+ve and 90 TTNtv-ve patients the proportions would be 0.25 and 0.75)

n = the total sample size

Φ is the standard Normal distribution function

Φ^{-1} is the standard Normal quantile function

α is Type I error

Genetic analysis

All potential participants underwent targeted next generation sequencing using the Illumina TruSight Cardio Sequencing kit or a custom Agilent SureSelect XT target capture with equivalent content, followed by sequencing on Illumina platforms or Life Technologies 5500XL. DNA libraries were prepared and sequenced according to manufacturers' protocols. Sequencing reads were aligned with BWA, and variants identified with GATK and annotated using the Ensembl Variant Effect Predictor as previously described[5]. TTNtv adjudicated likely pathogenic (as previously described[5] - minor allele frequency in the Exome Aggregation Consortium (ExAC) dataset <0.001 and impacting all principal cardiac transcripts) were confirmed by Sanger sequencing, or by review of mapped reads in Integrative Genomics Viewer.

In addition, variants in 39 other genes associated with inherited cardiac conditions[6] were analysed according to guidelines from the American College of Medical Genetics and Genomics & the (US) Association for Molecular Pathology[7] using the CardioClassifier decision support platform[8]. Variants adjudicated as Pathogenic or Likely Pathogenic after individual review are reported here. The 39 additional genes tested were as follows:

TNNT2, SCN5A, TCAP, MYH7, VCL, TPM1, TNNC1, RBM20, DSP, BAG3, LMNA, TPM1, MYBPC3, PRKAG2, TNNI3, MYL3, MYL2, ACTC1, CSRP3, PLN, TNNC1, GLA, FHL1, LAMP2, GAA, PKP2, DSG2, DSC2, JUP, RAF1, SOS1, PTPN11, KRAS, KCNQ1, KCNH2, KCNE1, KCNE2, RYR2, LDLR

CMR - Late gadolinium enhancement

For patients who underwent CMR, LGE images were acquired using a breath-hold inversion recovery sequence following administration of gadolinium contrast agent with inversion times optimised to null normal myocardium. Mid-wall myocardial fibrosis was recorded as present if detected in the primary and phase swapped image with cross cuts taken as appropriate. LGE was assessed by a CMR cardiologist, blinded to genotype.

Censoring

If a patient did not experience an event (n=90 for the primary outcome measure), data were right-censored on the day of their most recent device interrogation. In 91% of censored cases (13/15 TTNtv +ve, 69/75 TTNtv -ve), censoring was due to patients coming to the natural end of the study. As patients were followed-up from the time of their device implantation until their most recent device interrogation (up to 9 years post-implant), patients contributed differing amounts of time to the study. E.g. a patient having an ICD fitted in Jan 2016, whose most recent device interrogation was in Jan 2017 would be censored at 1 year, whereas a patient whose device was inserted in Jan 2012 whose most recent interrogation was on the same day would be censored at 5 years. All such patients were still alive and being actively followed-up clinically and by the Biobank research team – i.e .had not been lost to follow-up.

For the remaining 9% of censored cases, 6 patients were censored due to death (2/15 TTNtv +ve, 4/75 TTNtv -ve), 1 due to heart transplantation (0/15 TTNtv +ve, 1/75 TTNtv -ve) and 1 was lost to follow-up (0/15 TTNtv +ve, 1/75 TTNtv -ve). In these 8 cases, the exact time of censoring was the date of the device interrogation closest to death, transplant or loss to follow-up.

© 2019 Corden B et al. *JAMA Network Open.*

eAppendix 2. Additional Results

Genetic Analysis

Details of the pathogenic/likely pathogenic TTN_{tv} in the cohort are given in eTable 3.

Thirty-nine other core interpretable inherited cardiac disease genes were also asessed[16], and two likely-pathogenic/pathogenic variants were identified – an inframe deletion in *TNNT2* (found in a patient also carrying a TTN_{tv}) and a predicted truncating variant in *DSP* (found in a patient with no other likely pathogenic variant) (eTable 4).

Other predictors of ICD therapy

Statistically significant associations of ICD therapy were a history of NSVT prior to implant (HR=4.7, 95% CI 1.6-13.8, P=0.001), device type (ICD versus CRT-D, HR=2.7, 95% CI 1.3-5.8, P=0.012) and male sex (HR = 2.9, 95% CI 1.1-7.7, P=0.017).

Analysis of recurrent events

The mean number of episodes of appropriate ICD therapy was 0.37 per 5 years follow-up for the TTNtv-ve group, versus 1.03 per 5 years follow-up in the TTNtv+ve group. The association of TTNtv with the total number of ICD events was assessed in a frailty model and was statistically significant (HR = 2.92, 95% CI 1.02-8.3, P = 0.04).

Patients with and without LGE on CMR

If the primary analysis is restricted to those patients with LGE on CMR, the HR for the association of TTNtv and ICD therapy is 9.0 (95% CI 2.3 – 35), P = 0.002. For those with no LGE on CMR the HR is 6.3 but does not reach statistical significance (95% CI 0.9 – 46, P = 0.07).

TTNtv variants in the A band

Of the TTNtv variants, 19/28 (68%) were in the A-band (see eTable 3). Patients with a TTNtv in the A-band were not more likely to receive an ICD therapy than those with variants in other regions (HR=1.3, 95% CI 0.23-2.6, P=0.67).

Family history of SCD

Self-reported family history data for sudden death (but not specifically for confirmed arrhythmia) was available for a sub-set of patients (n=68, 16 TTNtv +ve). For this cohort, 6/16 TTNtv+ve patients reported a family history of sudden cardiac death (38%) compared to 13/52 TTNtv-ve patients (25%) – OR = 1.8 (95% CI 0.44 – 6.8), p=0.35. In this small cohort, a family history of SCD did not predict time to first appropriate ICD therapy, though the confidence interval was wide (HR=1.98, 95% CI 0.66-5.91, p=0.21). In a Cox PH model containing 'family history of SCD' and 'TTNtv status', only 'TTNtv status' significantly predicted appropriate ICD therapy (HR=6.4, p=0.002). It should be noted that these analyses are likely to be underpowered given the small sample size of 68 patients and should therefore be interpreted with caution. However, for reference these results have been added to the supplementary material.
CI 0.62-5.6, P=0.27). However, given the low number of patients with available family history data, this analysis is limited in power and should be interpreted with caution.

© 2019 Corden B et al. *JAMA Network Open.*

Tipping Point Analysis – findings are robust to extreme results in missing ICD data

There were 9 TTNtv +ve and 22 TTNtv -ve patients with missing ICD data due to missing or destroyed records. A tipping point analysis was conducted to assess how extreme the results would have to be amongst the missing data to tip the results into non-significance[9]. Online-only Figure 4 shows the results of this analysis. In summary, of the TTNtv +ve patients with ICD data, 13/28 (46%) experienced one or more appropriate ICD therapies, compared to 14/89 (16%) of TTNtv -ve patients (Fisher's exact test: OR = 4.6, P = 0.0016). If *zero* of the TTNtv +ve patients with missing ICD data had experienced an event (an extreme result given the fact that 46% of those with ICD data did have an event) then 6/22 (27%) of the TTNtv -ve patients with missing data would have to have experienced an event before the result tipped into non-significance – 1.7x the number that would be predicted to occur based on the proportion of events observed in the non-missing data. If 46% of the TTNtv +ve patients with missing data experienced an event (the number predicted based on the observed data) then 16/22 (73%) of the TTNtv -ve patients with missing data would have to experience an event – 4.6x the number expected.

© 2019 Corden B et al. *JAMA Network Open.*

eFigure 1. Tipping-Point Analysis for the Association of Titin-Truncating Variants With Appropriate Implanted Cardioverter Defibrillator Therapy Is Robust to Extreme Results Amongst Patients With Missing Implanted Cardioverter Defibrillator Data

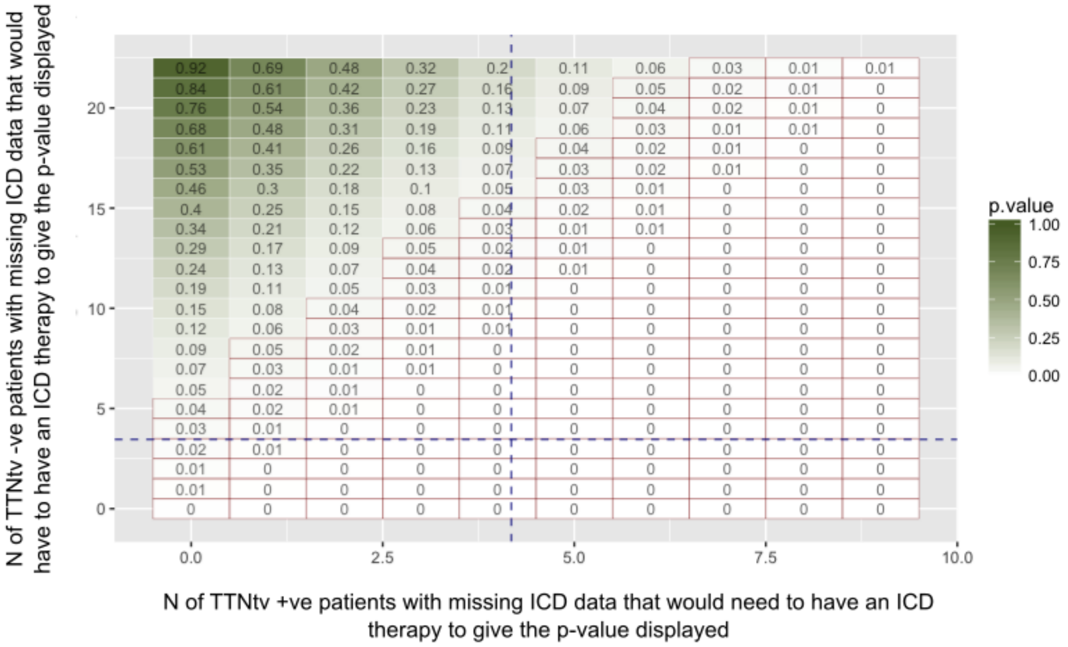

Heat map showing P-values in a Fisher's exact test for all potential combinations of results amongst the 31 patients (9 TTNtv, 22 TTNtv -ve) with missing ICD data, should this data be available and added to the already observed results.

eFigure 2. Association of Titin-Truncating Variants With an Increased Risk of Implanted Cardioverter Defibrillator Therapy in Analysis Restricted to Patients in the Primary Prevention Cohort

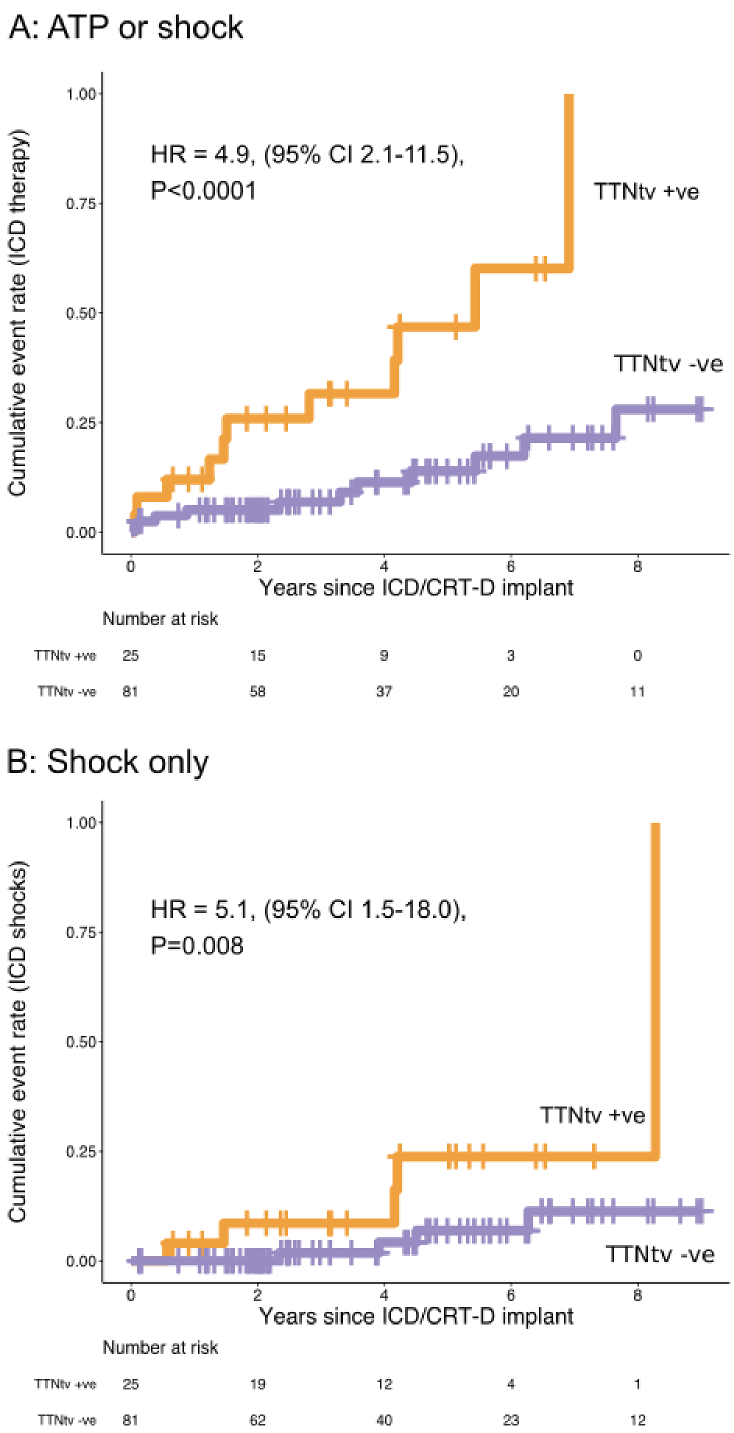

(A) Kaplan-Meier event curve for appropriate ICD therapy (anti-tachycardia pacing or shock) for VT >200bpm (as per MADIT-RIT high-rate therapy group[4]) or VF. (B) Kaplan-Meier event curve for appropriate ICD shock for VT >200bpm or VF. Data are restricted to primary prevention patients (secondary prevention patients, n=11, excluded). Statistical differences between the event curves are assessed by log-rank tests.

© 2019 Corden B et al. *JAMA Network Open.*

eFigure 3. No Significant Difference in Inappropriate Implanted Cardioverter Defibrillator Therapy Among Patients With Titin-Truncating Variants vs Those Without

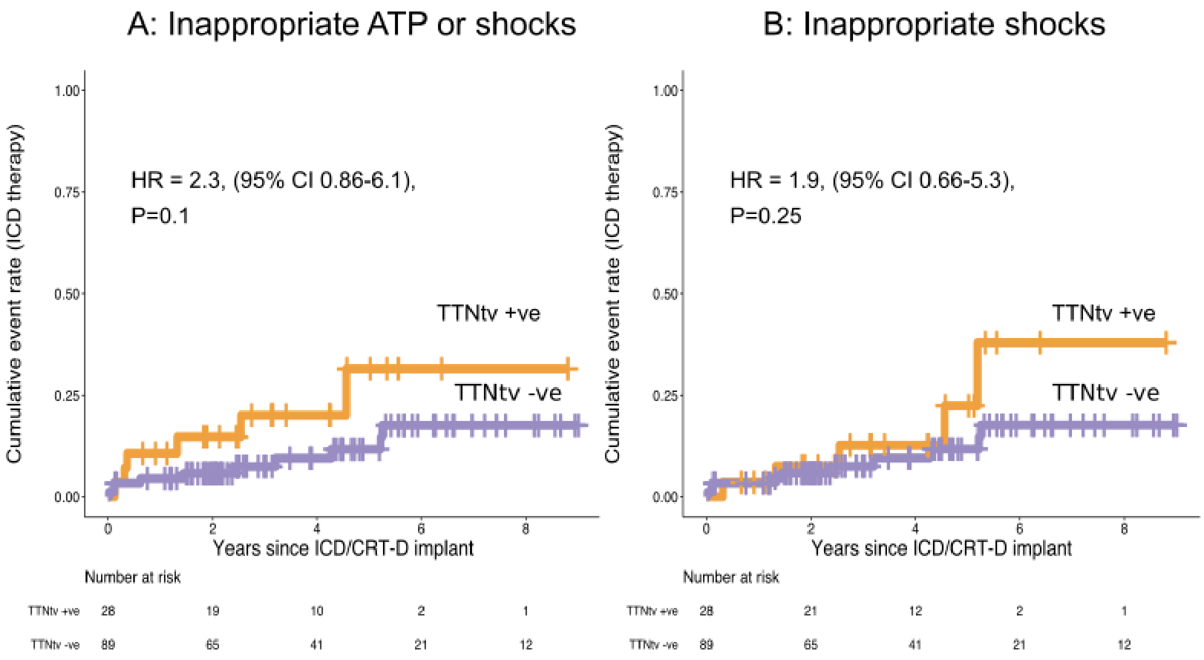

Kaplan-Meier event curves for (A) time to first inappropriate ICD therapy (ATP or shock) and (B) time to first inappropriate ICD shock

© 2019 Corden B et al. *JAMA Network Open.*

eTable 1. Baseline Characteristics of Patients in the Study Cohort vs Those Excluded Owing to Incomplete or Missing Arrhythmia Data

	TTNtv +ve			TTNtv -ve		
**Characteristic**	**Arrhythmia Data Available**<br>(N=28)<br>(N=21 for CMR, N=7 for echo)	**Arrhythmia Data Not Available**<br>(N=9)<br>(N=3 for CMR, N=6 for echo)	**p**	**Arrhythmia Data Available**<br>(N=89)<br>(N=69 for CMR, N=20 for echo)	**Arrhythmia Data Not Available**<br>(N=22)<br>(N=14 for CMR, N =8 for echo)	**p**
**Mean age at implant (SD) – yrs**	51.1 (10.8)	53.9 (17.9)	0.58	58.7 (12.5)	62.2(13.7)	0.26
**Male sex – no. (%)**	24 (86)	8 (89)	1	52 (58)	17 (77)	0.14
**Mean CMR LVEF (SD) - %**	31.2 (10.7)	27.5 (8.5)	0.52	30.5 (10.2)	35.4 (10.5)	0.11
**Mean CMR RVEF (SD) - %**	45.4 (13.6)	39.7 (14.2)	0.51	48 (16.2)	49.7 (16.1)	0.73
**Mean CMR LVEDV/BSA (SD) – [illegible]**	140.2 (31.1)	123.1 (44.2)	0.35	144 (36.6)	147.4 (50.4)	0.76
**Mean CMR RVEDV/BSA (SD) – [illegible]**	87.4 (28.6)	69.6 (21.3)	0.32	85.3 (28.6)	98.7 (27.3)	0.12
**Midwall LGE – no. (%)**	13 (62)	0/1 (0)*	0.41	28 (41)	6/10 (60)*	0.31
**Mean echo LVEF (SD) - %**	27.0 (10.6)	27.6 (9.6)	0.92	26.1 (9.2)	25.8 (9.7)	0.94
**Mean echo LVEDD/BSA (SD) – [illegible]**	31.6 (5.1)	31.4 (4.1)	0.87	34.1 (4.7)	33.8 (5.5)	0.82
**CRT – no. (%)**	11 (39)	6(67)	0.25	65 (73)	14(64)	0.43
**Mean BMI (SD) – kg/m²**	28.4 (6.3)	28.0 (4.4)	0.84	27.9 (5.5)	26.6 (5.2)	0.34
**Race – no. (%)**						
**Caucasian**	25 (89)	6 (67)	0.14	77 (87)	20 (91)	0.73
**Black**	2 (7)	0 (0)	1	4 (4)	0 (0)	0.58
**Asian**	1 (4)	2 (22)	0.14	5 (6)	2 (9)	0.62
**Other/Mixed**	0 (0)	1 (11)	0.24	3 (3)	0 (0)	1

Categorical variables compared with Fisher's exact tests, continuous variables with t-tests or Mann-Whitney tests. CMR data shown for those whose DCM diagnosis was confirmed by CMR, echo data for those diagnosed via echocardiography. BSA indicates body surface area, CMR cardiovascular magnetic resonance, CRT cardiac resynchronisation therapy, EDD end-diastolic diameter, EDV end-diastolic volume, EF ejection fraction, LGE late gadolinium enhancement, LV left ventricle. * LGE unavailable for 2 of 3 TTNtv+ve patients with CMR and 4 of 14 TTNtv-ve patients with CMR.

© 2019 Corden B et al. *JAMA Network Open.*

eTable 2. Details of Likely Pathogenic and Pathogenic Titin-Truncating Variants in the Cohort

cDNA variant[a]	Variant Type	Exon[b]	Band	Percent spliced in	allele count in ExAC	allele frequency in ExAC
c.82240C>T	Nonsense	327	A-band	100	2	0.0000166
c.98506C>T	Nonsense	353	A-band	100	0	0
c.58732+2T>C	Essential splice site	299	A-band	100	0	0
c.93291_93301delTGTTGGTGAGC	Frameshift	340	A-band	100	0	0
c.63025C>T	Nonsense	305	A-band	100	0	0
c.76666_76684dupATAATTGATGTCACTAGCA	Frameshift	327	A-band	100	0	0
c.1489G>T	Nonsense	9	Z-disk	100	0	0
c.3380+1G>C	Essential splice site	20	Near Z-disk	100	0	0
c.44364delC	Frameshift	241	I-band	100	0	0
c.76355G>A	Nonsense	327	A-band	100	0	0
c.90567_90573delGAGTATT	Frameshift	336	A-band	100	0	0
c.41473C>T	Nonsense	227	I-band	100	0	0
c.67567delG	Frameshift	320	A-band	100	0	0
c.69705delA	Frameshift	326	A-band	100	0	0
c.63025C>T	Nonsense	305	A-band	100	0	0
c.89216delC	Frameshift	335	A-band	100	0	0
c.100267_100268delAA	Frameshift	358	A-band	99	0	0
c.8437dupG	Frameshift	36	I-band	100	0	0
c.86107C>T	Nonsense	327	A-band	100	0	0
c.9448C>T	Nonsense	40	I-band	100	2	0.0000166
c.73846C>T	Nonsense	327	A-band	100	0	0
c.97492+1G>A	Essential splice site	350	A-band	99	0	0
c.100390G>T	Nonsense	358	A-band	99	0	0
c.102958delA	Frameshift	359	M-band	100	0	0
c.53881+1G>T	Essential splice site	280	A-band	100	0	0
c.51781C>T	Nonsense	274	A-band	100	0	0
c.43602_43615delGCGCCTACACACCA	Frameshift	237	I-band	100	0	0
c.45307C>T	Nonsense	246	I-band	100	0	0

[a] described with respect to transcript LRG_391t1

[b] exons numbered according to locus reference genomic numbering (LRG_391)

ExAC = exome aggregation consortium (exac.broadinstitute.org)

eTable 3. Details of Likely Pathogenic and Pathogenic Variants Found in the Cohort Other Than Titin-Truncating Variants in the *TTN* Gene

Gene	cDNA variant	Variant type	ACMG rules activated	ExAC count (frequency)
TTNT2	c.629_631delAGA	Inframe_Indel	PP1_strong, PS4,PM2,PM4	0 (0)
DSP	c.3928A>T	Nonsense	PVS1,PM2	0 (0)

© 2019 Corden B et al. *JAMA Network Open.*

eTable 4. Cox Proportional Hazards Model for Time to First Appropriate Implanted Cardioverter Defibrillator Therapy Among the Whole Cohort

Variable	Adjusted HR	Lower 95% CI	Upper 95% CI	P
Age (per 5 yrs)	1.1	0.89	1.3	0.44
Sex (Male vs Female)	1.8	0.63	5.0	0.28
Device type (ICD vs CRT-D)	2.0	0.84	4.7	0.12
LBBB	0.92	0.34	2.5	0.87
TTNtv (+ve vs –ve)	3.5	1.3	9.5	0.01

Cox proportional hazards model for time to first appropriate ICD therapy (ATP or shocks). TTNtv remains associated with ICD therapy after adjustment for age, sex, device type and presence of LBBB (variables which differ between the TTNtv +ve and –ve groups at baseline).

eTable 5. Cox Proportional Hazards Model for Time to First Appropriate Implanted Cardioverter Defibrillator Therapy Among the Primary Prevention Cohort

Variable	Adjusted HR	Lower 95% CI	Upper 95% CI	P
Age (per 5 yrs)	1.1	0.90	1.4	0.36
Sex (Male vs Female)	2.2	0.67	7.3	0.19
Device type (ICD vs CRT-D)	1.8	0.64	5.1	0.27
LBBB	0.98	0.30	3.2	0.97
TTNtv (+ve vs –ve)	3.6	1.1	11.7	0.03

Cox proportional hazards model for time to first appropriate ICD therapy (ATP or shocks). Primary prevention cohort (secondary prevention patients, n=11, excluded). TTNtv remains associated with ICD therapy after adjustment for age, sex, device type and presence of LBBB (variables which differ between the TTNtv +ve and –ve groups at baseline).

eTable 6. Cox Proportional Hazards Model for Time to First Appropriate Implanted Cardioverter Defibrillator Therapy Among the Whole Cohort With Adjustment for Body Mass Index and Left Ventricle Ejection Fraction

Variable	Adjusted HR	Lower 95% CI	Upper 95% CI	P
Age (per 5 yrs)	1.1	0.89	1.3	0.42
Sex (Male vs Female)	1.8	0.64	5.1	0.27
Device type (ICD vs CRT-D)	2.1	0.86	5.2	0.10
LBBB	0.90	0.34	2.4	0.84
LV Ejection Fraction (per 5%)	0.90	0.75	1.08	0.26
Body Mass Index	0.99	0.92	1.07	0.81
TTNtv (+ve vs –ve)	3.6	1.3	9.5	0.01

Cox proportional hazards model for time to first appropriate ICD therapy (ATP or shocks). TTNtv remains associated with ICD therapy after adjustment for age, sex, device type, presence of LBBB (variables which differ between the TTNtv +ve and –ve groups at baseline) and also Body Mass Index (BMI) and LV ejection fraction.

© 2019 Corden B et al. *JAMA Network Open.*

eTable 7. Cox Proportional Hazards Model for Time to First Appropriate Implanted Cardioverter Defibrillator Therapy Among the Primary Prevention Cohort With Adjustment for Body Mass Index and Left Ventricle Ejection Fraction

Variable	Adjusted HR	Lower 95% CI	Upper 95% CI	P
Age (per 5 yrs)	1.1	0.89	1.4	0.36
Sex (Male vs Female)	2.2	0.67	7.5	0.19
Device type (ICD vs CRT-D)	1.8	0.63	5.3	0.27
LBBB	0.98	0.29	3.3	0.97
LV Ejection Fraction (per 5%)	0.92	0.76	1.1	0.44
Body Mass Index	0.97	0.90	1.1	0.47
TTNtv (+ve vs –ve)	3.9	1.2	12.8	0.03

Cox proportional hazards model for time to first appropriate ICD therapy (ATP or shocks). Primary prevention cohort (secondary prevention patients, n=11, excluded). TTNtv remains associated with ICD therapy after adjustment for age, sex, device type, presence of LBBB (variables which differ between the TTNtv +ve and –ve groups at baseline) and also Body Mass Index (BMI) and LV ejection fraction.

eTable 8: Cox Proportional Hazards Model for Time to First Appropriate Implanted Cardioverter Defibrillator Therapy Among the Whole Cohort With Adjustment for History of Nonsustained Ventricular Tachycardia

Variable	Adjusted HR	Lower 95% CI	Upper 95% CI	P
Age (per 5 yrs)	1.1	0.91	1.4	0.30
Sex (Male vs Female)	2.5	0.78	7.9	0.12
Device type (ICD vs CRT-D)	1.9	0.67	5.5	0.22
LBBB	1.3	0.40	4.0	0.70
LV Ejection Fraction (per 5%)	0.92	0.76	1.1	0.35
Body Mass Index	0.99	0.92	1.1	0.83
History of NSVT	3.2	1.02	10.0	0.046
TTNtv (+ve vs –ve)	3.1	1.1	9.2	0.04

As for ETable 3 but with additional adjustment for history of NSVT prior to device implant. Both TTNtv and a prior history of NSVT are independently associated with a risk of appropriate ICD therapy.

eTable 9. Cox Proportional Hazards Model for Time to First Appropriate Shock Among the Whole Cohort

Variable	Adjusted HR	Lower 95% CI	Upper 95% CI	P
Age (per 5 yrs)	1.1	0.75	1.5	0.70
Sex (Male vs Female)	4.6	0.54	38.2	0.16
Device type (ICD vs CRT-D)	2.0	0.46	9.1	0.35
LBBB	1.1	0.24	5.4	0.88
LV Ejection Fraction (per 5%)	1.1	0.83	1.5	0.51
Body Mass Index	1.03	0.93	1.1	0.54
TTNtv (+ve vs –ve)	2.3	0.52	10.4	0.27

As for ETable 3 but for time to appropriate shock only.

eTable 10. Cox Proportional Hazards Model for Time to First Appropriate Shock Among the Primary Prevention Cohort

Variable	Adjusted HR	Lower 95% CI	Upper 95% CI	P
Age (per 5 yrs)	1.1	0.78	1.6	0.52
Sex (Male vs Female)	4.1	0.45	37.6	0.21
Device type (ICD vs CRT-D)	2.4	0.43	13.0	0.32
LBBB	1.2	0.17	8,4	0.86
LV Ejection Fraction (per 5%)	1.06	0.80	1.4	0.70
Body Mass Index	1.02	0.92	1.1	0.77
TTNtv (+ve vs –ve)	3.1	0.46	21.0	0.26

As for ETable 4 but for time to appropriate shock only.

eTable 11: Cox Proportional Hazards Model for Time to First Appropriate Shock With Adjustment for History of Nonsustained Ventricular Tachycardia

Variable	Adjusted HR	Lower 95% CI	Upper 95% CI	P
Age (per 5 yrs)	1.1	0.76	1.7	0.56
Sex (Male vs Female)	5.2	0.63	43.4	0.13
Device type (ICD vs CRT-D)	2.4	0.43	13.6	0.32
LBBB	2.7	0.42	17.7	0.30
LV Ejection Fraction (per 5%)	1.1	0.81	1.4	0.67
Body Mass Index	1.03	0.93	1.1	0.63
History of NSVT	6.0	0.65	55.1	0.11
TTNtv (+ve vs –ve)	3.0	0.54	17.1	0.21

As for ETable 8, but with additional adjustment for history of NSVT prior to implant.

© 2019 Corden B et al. *JAMA Network Open.*

eTable 12. Baseline Characteristics of Patients with Cardiovascular Magnetic Resonance Data vs Those Without

Characteristic		Echo group (N=27)	CMR group (N=90)	P-value
**Mean age at implant (SD) – yrs**		57.8 (12.4)	56.7 (12.6)	0.68
**Male sex – no. (%)**		21 (78)	55 (61)	0.17
**Primary prevention indication – no. (%)**		23 (85)	83 (92)	0.27
**Median follow-up (IQR) – yrs**		6.4 (6.4)	3.3 (3.6)	0.001
**Median time from diagnosis to device implant (IQR) yryrs**		1.3 (4.4)	0.9 (4.0)	0.47
**LBBB at implant – no. (%)**		13 (48)	51 (57)	0.51
**CRT – no. (%)**		18 (67)	58 (64)	1
**Mean BMI (SD) – kg/m^2**		27.9 (6.1)	28.1(5.5)	0.88
**Race – no. (%)**				
	**Caucasian**	24 (89)	78 (87)	1
	**Black**	2 (7)	4 (4)	0.62
	**Asian**	1 (4)	5 (6)	1
	**Other/Mixed**	0 (0)	3 (3)	1
**Medications – no. (%)**				
	**ACE inhibitor/ARB**	27 (100)	89 (99)	1
	**Beta-blocker**	20 (74)	77 (86)	0.24
	**Mineralocorticoid-receptor antagonist**	18 (67)	63 (70)	0.81
	**Amiodarone**	9 (33)	16 (18)	0.11

Categorical variables compared with Fisher's exact tests, continuous variables with t-tests or Mann-Whitney tests. CMR data shown for those whose DCM diagnosis was confirmed by CMR, echo data for those diagnosed via echocardiography. BSA indicates body surface area, CMR cardiovascular magnetic resonance, CRT cardiac resynchronisation therapy, EDD end-diastolic diameter, EDV end-diastolic volume, EF ejection fraction, LBBB left bundle branch block, LGE late gadolinium enhancement, LV left ventricle.

© 2019 Corden B et al. *JAMA Network Open.*

eTable 13: Cox Proportional Hazards Model for Time to First Appropriate Implanted Cardioverter Defibrillator Therapy Among the Primary Prevention Patient Cohort With Adjustment for Midwall Fibrosis as Determined by Late-Gadolinium Enhancement on Cardiovascular Magnetic Resonance Images

Variable	Adjusted HR	Lower 95% CI	Upper 95% CI	P
Age (per 5 yrs)	1.1	0.83	1.5	0.52
Sex (Male vs Female)	0.81	0.16	4.1	0.80
Device type (ICD vs CRT-D)	1.1	0.23	4.9	0.93
LBBB	1.1	0.19	6.2	0.94
LV Ejection Fraction (per 5%)	0.87	0.68	1.1	0.29
Body Mass Index	0.96	0.87	1.1	0.46
Midwall LGE (present vs absent)	1.8	0.43	7.5	0.42
TTNtv (+ve vs –ve)	13.3	2.2	79.9	0.005

As for ETable 11 but with exclusion of secondary prevention patients.

© 2019 Corden B et al. *JAMA Network Open.*

eReferences

1. Chow SS, J; Wang, H. *Sample Size Calculations in Clinical Research*. 2nd ed: Chapman & Hall; 2008.
2. Ellenbogen KA, Levine JH, Berger RD, Daubert JP, Winters SL, Greenstein E, Shalaby A, Schaechter A, Subacius H, Kadish A, Defibrillators in Non-Ischemic Cardiomyopathy Treatment Evaluation I. Are implantable cardioverter defibrillator shocks a surrogate for sudden cardiac death in patients with nonischemic cardiomyopathy? Circulation 2006;**113**(6):776-82.
3. Kober L, Thune JJ, Nielsen JC, Haarbo J, Videbaek L, Korup E, Jensen G, Hildebrandt P, Steffensen FH, Bruun NE, Eiskjaer H, Brandes A, Thogersen AM, Gustafsson F, Egstrup K, Videbaek R, Hassager C, Svendsen JH, Hofsten DE, Torp-Pedersen C, Pehrson S, Investigators D. Defibrillator Implantation in Patients with Nonischemic Systolic Heart Failure. N Engl J Med 2016;**375**(13):1221-30.
4. Moss AJ, Schuger C, Beck CA, Brown MW, Cannom DS, Daubert JP, Estes NA, 3rd, Greenberg H, Hall WJ, Huang DT, Kautzner J, Klein H, McNitt S, Olshansky B, Shoda M, Wilber D, Zareba W, Investigators M-RT. Reduction in inappropriate therapy and mortality through ICD programming. N Engl J Med 2012;**367**(24):2275-83.
5. Roberts AM, Ware JS, Herman DS, Schafer S, Baksi J, Bick AG, Buchan RJ, Walsh R, John S, Wilkinson S, Mazzarotto F, Felkin LE, Gong S, MacArthur JA, Cunningham F, Flannick J, Gabriel SB, Altshuler DM, Macdonald PS, Heinig M, Keogh AM, Hayward CS, Banner NR, Pennell DJ, O'Regan DP, San TR, de Marvao A, Dawes TJ, Gulati A, Birks EJ, Yacoub MH, Radke M, Gotthardt M, Wilson JG, O'Donnell CJ, Prasad SK, Barton PJ, Fatkin D, Hubner N, Seidman JG, Seidman CE, Cook SA. Integrated allelic, transcriptional, and phenomic dissection of the cardiac effects of titin truncations in health and disease. Sci Transl Med 2015;**7**(270):270ra6.
6. Walsh R, Thomson KL, Ware JS, Funke BH, Woodley J, McGuire KJ, Mazzarotto F, Blair E, Seller A, Taylor JC, Minikel EV, Exome Aggregation C, MacArthur DG, Farrall M, Cook SA, Watkins H. Reassessment of Mendelian gene pathogenicity using 7,855 cardiomyopathy cases and 60,706 reference samples. Genet Med 2017;**19**(2):192-203.
7. Richards S, Aziz N, Bale S, Bick D, Das S, Gastier-Foster J, Grody WW, Hegde M, Lyon E, Spector E, Voelkerding K, Rehm HL, Committee ALQA. Standards and guidelines for the interpretation of sequence variants: a joint consensus recommendation of the American College of Medical Genetics and Genomics and the Association for Molecular Pathology. Genet Med 2015;**17**(5):405-24.
8. Whiffin N, Walsh R, Govind R, Edwards M, Ahmad M, Zhang X, Tayal U, Buchan R, Midwinter W, Wilk AE, Najgebauer H, Francis C, Wilkinson S, Monk T, Brett L, O'Regan DP, Prasad SK, Morris-Rosendahl DJ, Barton PJR, Edwards E, Ware JS, Cook SA. CardioClassifier: disease- and gene-specific computational decision support for clinical genome interpretation. Genet Med 2018.
9. Liublinska V, Rubin DB. Sensitivity analysis for a partially missing binary outcome in a two-arm randomized clinical trial. Stat Med 2014;**33**(24):4170-85.

© 2019 Corden B et al. *JAMA Network Open.*
